# The architecture of mental health: identifying the combination of apartment building design requirements for positive mental health outcomes

**DOI:** 10.1016/j.lanwpc.2023.100807

**Published:** 2023-06-22

**Authors:** Paula Hooper, Alexandra Kleeman, Nicole Edwards, Julian Bolleter, Sarah Foster

**Affiliations:** a Australian Urban Design Research Centre (AUDRC), School of Design, The University of Western Australia, Australia; b Centre for Urban Research, School of Global, Urban and Social Studies, RMIT University, Australia

**Keywords:** Housing, Apartments, High rise, Mental health, Policy, Evaluation, Design, Architecture

## Abstract

**Background:**

Housing quality is a crucial determinant of mental health. While the construction of high-rise buildings is a popular policy strategy for accommodating population growth in cities, there is considerable debate about the health consequences of living in poorly designed apartments. Drawing on three Australian state government apartment design policies introduced to improve apartment design quality, this study aimed to identify the combination of design requirements that were optimally supportive of positive mental health.

**Methods:**

K-means cluster analyses identified groups of buildings (*n =* 172) that were homogenous in their implementation of a mix of *n* = 80 measured design requirements. Positive mental health was measured using the Warwick–Edinburgh Mental Well-being Scale (WEMWBS). Linear mixed-effects models controlling for demographic characteristics, self-selection factors and clustering of participants within buildings compared residents in the different clusters.

**Findings:**

Residents in the *"high policy performance buildings",* characterised by having a greater implementation of *n =* 29 design requirements across nine design elements, had significantly higher (+1.96 points) WEMWBS scores compared with residents in the *"low policy performance buildings".*

**Interpretation:**

This study is the first to empirically identify a mix of policy-specific architecture design requirements that are associated with positive mental health in apartment residents. These findings provide vital empirical evidence to inform national and international apartment and high-rise housing policies, and design instruments and practices to protect people's health in apartment dwellings.

**Funding:**

The High Life project is funded by a 10.13039/501100000960Healthway Research Intervention Project grant (#31986) and an 10.13039/501100000923Australian Research Council (ARC), Discovery Early Career Researcher Award (DECRA) (DE160100140). NE is supported by an Australian Research Council (10.13039/501100000923ARC) Linkage Project (LP190100558). SF is supported by an Australian Research Council (10.13039/501100000923ARC) Future Fellowship (FT210100899).

**Research in context**

**Evidence before this study**

Previous research has shown that apartment housing quality is a crucial determinant of mental health. While the construction of high-rise buildings is a popular policy strategy for accommodating population growth in cities, there is still much debate about the health consequences of living in high-rise apartments. Australian cities are experiencing a significant boom in apartment construction that will leave a legacy for future generations. A significant critique of the current evidence base is a lack of policy and design specificity; housing studies have not evaluated design outcomes or health impacts of the policies or legislation that underpin the design and delivery of buildings. In addition, studies typically do not include detailed assessments of apartment design or adherence to policy and design standards. There is also an emerging shift in focus from housing characteristics that prevent discomfort, dissatisfaction, or disease, to how design can create conditions that foster positive outcomes and enhance occupants' social and emotional wellbeing.

**Added value of this study**

This study makes a crucial contribution to the literature. It is the first study to measure and examine the implementation and composition of design requirements (*n* = 80) derived from operational apartment design policies and their association with residents' positive mental health in a large sample of buildings and residents across three cities. It provides empirical evidence of a dose–response relationship between implementing a combination of *n* = 29 apartment policy design requirements and positive mental wellbeing. The results provide evidence to inform future architectural and design policy and advance practice for good apartment design and high-rise living that may benefit the mental health of occupants.

**Implications of all the available evidence**

Our results have practical implications as they identify the combination of design requirements that should be prioritised in building design and approval processes to promote optimal resident mental health outcomes. The findings reiterate the importance of architecture and design instruments that facilitate the implementation of minimum policy standards to guide architectural and urban design thinking, policy, and practice and, ultimately, the health of future high-rise housing stock. Moreover, these findings provide vital empirical evidence to inform national and international apartment and high-rise housing policy and design instruments and practices to protect the health of people living in apartments. The results can be used to advocate for the adoption (where currently missing) or retention (where presently included) of the specific design features identified in future design policies. Finally, this study addresses a significant gap in the literature, providing empirical evidence that apartment buildings that adhere to Australian apartment design policies have the potential to promote the positive mental wellbeing of the inhabitants.

## Introduction

Substandard housing is a significant public health issue.^1^ There is a well-developed international evidence base linking inadequate indoor air quality, space, natural ventilation, sunlight, acoustic and visual privacy, insulation, and thermal comfort with various physical health impacts, including asthma, hypertension, upper respiratory tract infections, poor sleep quality, and infectious and chronic diseases.^2^, ^3^, ^4^, ^5^ Moreover, the effects of prolonged exposure to poor quality housing and associated physical ill health can, in turn, have negative implications for the psychological health of residents.^6^

A number of cross-sectional studies from high-income countries with a focus on mental health outcomes have found that apartment residents have poorer mental health than residents in other housing typologies,^2^^,^^6^ and emerging evidence indicates that apartment design may play a role in this association. For example, insufficient space, restrictive layouts, low levels of sunlight and natural ventilation, and inability to control environmental stressors (e.g., acoustic and visual privacy) can adversely impact residents' quality of life, influence mental health through increasing anxiety and stress^2^^,^^4^^,^^6^^,^^7^ and have been associated with depressive symptoms and mental health issues.^2^ Higher density living may also impact mental health through social mechanisms^6^^,^^8^ with studies reporting that high-rise occupants experience increased loneliness or social isolation, negatively influencing mental wellbeing.^8^ A systematic review explored the collective evidence on the longitudinal impact of housing disadvantage in 12 temporally ordered studies where the exposure to housing disadvantage preceded mental health measures. Substandard housing quality was shown to be linked to higher stress levels, anxiety was higher in renters than owners and overcrowding was linked to a measure of mean depressive symptoms but not a depressive disorder.^9^ Another longitudinal study also reported a decrease in depressive symptoms and that improvements to the dwelling were related to improved mental health.^10^

However, definite conclusions cannot currently be drawn with the conflicting evidence due to differences in methodological approaches and substantial heterogeneity in how both the mental health outcome variable and housing quality was defined and measured.^2^^,^^9^^,^^11^

Internationally and within Australia, there is growing emphasis on compact city planning policies to help realise urban sustainability goals. As such, apartments are housing a rapidly increasing number of people. Nearly one-third (30.9%) of the almost one million new dwellings across Australia from 2016 to 2021 were apartments.^12^ This apartment boom has raised concerns about the potential negative impacts of poor design on apartment building residents, however few studies have examined apartment design and health in Australia.^4^^,^^5^ Most studies have been conducted in North America and Europe, where the public sector developed many high-rise residential buildings after the Second World War in deprived areas.^7^ This may partly account for the adverse mental health outcomes of apartment housing.^2^^,^^4^^,^^7^ Additionally, previous studies have compared living in high-rise buildings to other housing types, but have not considered differences in design quality or characteristics of varying high-rise buildings and apartments.

Studies exploring high-rise living or apartment design and mental health have typically relied on measures of negative mental health, such as psychological distress, that are indicative of impaired mental health and may reflect common mental disorders, like depressive and anxiety disorders,^6^ but at a level insufficient for the diagnosis of a disorder or psychiatric illness.^11^ However, there is increasing international interest in positive mental health that refers to the presence of positive emotions and good functioning, defined by The World Health Organisation as *"a state of wellbeing which allows individuals to realise their abilities, cope with the normal stresses of life, work productively and fruitfully, and make a contribution to their community",*^13^ p.13).

A significant critique of the current evidence base is a lack of policy and design specificity. Studies have rarely evaluated the design outcomes of the policies or legislation that underpin the design of the buildings studied and have not included detailed or direct assessments of apartment design or adherence to these policy and design standards.^14^ Barros et al., (2019)^8^ have called for a re-examination of housing policies from a multidisciplinary perspective that considers public health concerns together with more empirical research to guide evidence-informed design decisions. Moreover, there is an emerging shift in focus from housing characteristics that prevent discomfort or disease, to how design can create conditions conducive to positive outcomes that enhance occupants' physical, social and emotional wellbeing^15^ and an analysis of how apartment design can contribute to positive mental health.^8^

### The policy of apartment design

The boom in apartment construction in Australia has prompted the introduction of residential apartment design policies by state governments to regulate and improve design outcomes conducive to good amenity and wellbeing.^16^ For example, in 2002, the NSW state government introduced the State Environmental Planning Policy 65 (SEPP65) and Apartment Design Guide.^17^ Other Australian states have since followed suit: the Western Australian government introduced State Planning Policy 7.3 (SPP7.3) Residential Design Codes Volume 2 - Apartments in 2019^18^; and the Victorian state government introduced the Better Apartments Design Standards (BADS) in 2017.^19^ All three policies acknowledge apartment design's role in promoting health and wellbeing and include explicit aims to achieve this. Our previous analysis evaluated how well contemporary apartment buildings conformed with the respective state policies and identified the importance of a comprehensive design policy to achieving better apartment design and amenity.^3^ However, empirical research evaluating whether the implementation of these apartment design policies is associated with residents' health is rare^15^ and there is little understanding of which *combination* of apartment policy design requirements are optimal for promoting positive mental health outcomes for residents. Previous studies have used cluster analysis techniques to reveal neighbourhood 'types' with different combinations of built environment characteristics important for supporting physical activity behaviours.^20^ In our study context, this technique can be applied to characterise apartment building types and explore how different combinations of apartment design features impact residents' mental health and wellbeing.

This paper aimed to assess the performance and impact of apartment design policies in three Australian states by (1) characterising the apartment buildings based on the 'mix' or combination of policy design requirements implemented; and (2) exploring the combination of design requirements associated with the positive mental health of apartment residents.

## Methods

**The High Life project** is a cross-sectional study^14^ evaluating the implementation of the SEPP65, SPP7.3 and BADS apartment design policy requirements in apartment developments in Sydney, Perth and Melbourne and exploring the relationship between apartment design and residents' health and wellbeing outcomes.^14^

### Apartment building selection and participant recruitment

The building selection process has been described in full elsewhere.^3^^,^^14^ The sample comprises 172 apartment buildings with 40 or more apartments, three or more storeys and built between 2006 and 2016, that were randomly selected from the greater metropolitan areas of Sydney (*n* = 57), Melbourne (*n* = 46), and Perth (*n* = 69). Building residents completed a self-report survey about their apartment and building design and a range of physical, social and mental wellbeing outcomes and socio-demographics.^14^ Over a two-year period (2017–2019), 10,560 apartment households were contacted via post and invited to participate in the study survey. Accounting for a 5% rental vacancy rate, the overall response rate for the survey was 13.2%. The analytic sample for this study after excluding participants with missing data was 1135. The High Life Study was approved by the RMIT University Design and Social Context College Human Ethics Advisory Network (CHEAN B 21146-10/17).

### Measuring apartment design requirements

Each state government policy, SEPP65 (NSW), SPP7.3 (WA) and BADS (VIC), was reviewed for design requirements that could plausibly impact positive health and wellbeing outcomes across eight design themes: (1) solar and daylight access; (2) natural ventilation; (3) acoustic privacy; (4) outlook and visual privacy; (5) indoor space; (6) private outdoor space; (7) communal outdoor space; and (8) circulation spaces (i.e., corridors and foyers). These design elements were derived from prior research that audited apartment design policies for their potential to promote health^16^ and were broadly consistent with the groupings of design requirements in the three policies. Design requirements that impacted the ease and experience of apartment living were also extracted: (9) bicycle and car parking; and (10) apartment mix. Design requirements were eligible if they included a stated and potentially measurable criterion or standard relating to the design of the apartment, residential floors, or building.^21^

To assess the implementation of each design requirement in the apartment buildings, tailored measures were created using architectural or development plans (including floor plates for each building level and elevations for each aspect) sourced from development applications for the apartment buildings.^14^^,^^21^ Where buildings were sited within the same apartment complex, the communal space measures for the complex were assigned to all buildings in that complex. Architecturally qualified research assistants extracted data for each requirement. The measures were developed with guidance from a stakeholder panel comprised of professional architects and urban designers from the Department of Planning Lands and Heritage and the Western Australian Office of the Government Architect. Data were extracted and measures computed for 10,533 residential apartments and 1094 residential floors within the 172 buildings.^3^ For this study, the implementation of the requirements was summarised at the building level (e.g., percentage of apartments in the building with one aspect). Table 1 outlines the *n* = 80 design requirement measures examined. All buildings were assessed against the complete pool of design requirements derived from the three policies, regardless of whether the requirement/standard applied in the state.

**Table 1:** Apartment building design requirements from the SEPP65 (NSW), SPP7.3 (WA) and BADS (VIC) apartment design policies.

Design requirement		NSW – SEPP65	WA – SPP7.3	VIC - BADS	Building type	Solar/daylight	Natural ventilation	Indoor space & layout	Private open space	Communal space	Circulation space	Acoustic privacy	Outlook/visual privacy	Parking	Apartment mix
1)	Number of residential buildings within the complex	✓	✓	✓	•										
2)	Number of storeys/floors - counted from the ground floor and above	✓	✓	✓	•										
3)	Number of apartments within the building	✓	✓	✓	•										
4)	Plot Ratio (the ratio of the total floor area of a building to the area of the site)	✓	✓	✓	•										
5)	% of apartments with (only) 1 aspect	✓	✓			•									
6)	% of apartments with 2 aspects	✓	✓			•									
7)	% of apartments with 3 aspects	✓	✓			•									
8)	% of apartments where the main aspect gets ≥2 h of direct sunlight every day	✓	✓			•									
9)	% of apartments with a main northerly aspect	✓	✓	✓		•									
10)	% of apartments where all habitable rooms have a window in an external wall	✓	✓	✓		•	•	•							
11)	% of apartments with a ratio of the openable living room window area to open plan floor area ≥5%	✓				•	•	•							
12)	% of apartments with a ratio of the living room window area to open plan floor area ≥10%	✓	✓			•	•	•							
13)	% of apartments with all bedrooms + living areas located on an external face/wall	✓	✓					•							
14)	% of apartments where the habitable room depths are ≤2.5 × ceiling height (≤3 for open plan)	✓	✓			•	•	•							
15)	% of apartments with a living area depth ≤8 m	✓					•	•							
16)	% of apartments with a total depth ≤18 m	✓					•								
17)	% of apartments with the face/aspect on which the living area of the apartment is located is ≥12 m from the site boundary	✓										•	•		
18)	% of apartments that are naturally cross ventilated with windows on two perpendicular walls	✓	✓	✓			•								
19)	% of apartments with a correct building street setback distance (3m) from the face of the building to the road centreline		✓									•	•		
20)	% of apartments with private storage external to the apartment	✓	✓	✓				•							
21)	% of apartments with the area of the main/1st bedroom ≥10 m^2^	✓	✓					•							
22)	% of apartments with the area of the 2nd, 3rd or 4th bedroom ≥9 m^2^	✓	✓					•							
23)	% of apartments with the width/depth dimensions of the main/1st bedroom ≥3 m	✓	✓					•							
24)	% of apartments with the width/depth of the 2nd, 3rd or 4th bedroom ≥3 m	✓	✓	✓				•							
25)	% of apartments with a dedicated study room		✓	✓				•							
26)	% of apartments with a dedicated laundry room		✓	✓				•							
27)	% of apartments meeting the minimum internal floor area: Studio = 35 m^2^; 1-bed = 50 m^2^; 2-bed = 70 m^2^; 3-bed = 90 m^2^ + 5 m^2^ 2nd + bathrooms; 4-bed = 102 m^2^ + 5 m^2^ 2nd + bathrooms	✓						•							
28)	% of apartments meeting the minimum internal floor area size: Studio = 37 m^2^; 1-bed = 47 m^2^; 2-bed & 1-bath = 67 m^2^; 3-bed & 1-bath = 90 m^2^ (+3 m^2^ for 2nd/separate toilet, 5 m^2^ for a 2nd bathroom, 9 m^2^ per extra bedroom)		✓					•							
29)	% of apartments with any private open (outdoor) space	✓	✓	✓					•						
30)	% of apartments with a private outdoor courtyard	✓	✓	✓					•						
31)	% of apartments with a private balcony	✓	✓	✓					•						
32)	% of apartments with the 1st/main balcony or courtyard accessible from the living area	✓	✓	✓					•						
33)	% of apartments with a balcony depth less than the width (i.e., long side facing out)	✓							•						
34)	% of apartments with a courtyard depth less than the width (i.e., long side facing out)	✓							•						
35)	% of apartments minimum primary balcony size (area) requirement: Studio ≥4 m^2^; 1-bedroom ≥8 m^2^; 2-bedroom ≥10 m^2^; 3-bedroom ≥12 m^2^ (SEPP65/SPP7.3)	✓	✓						•						
36)	% of apartments minimum primary balcony size (area) requirement: Studio, 1- and 2-bedroom ≥8 m^2^; 3+ bedroom dwelling ≥12 m^2^; Courtyards ≥25 m^2^ (BADS)			✓					•						
37)	% of apartments that meet the minimum courtyard size (area) requirement (15 m^2^) (SEPP65/SPP7.3)	✓	✓						•						
38)	% of apartments that meet the minimum primary balcony depth requirement: 1-bedroom ≥2 m; 2-bedroom ≥2 m; 3-bedroom ≥2.4 m (SEPP65/SPP7.3)	✓	✓						•						
39)	% of apartments that meet the minimum primary balcony depth requirement: studio or 1-bedroom ≥1.8 m; 2-bedroom dwelling ≥2 m; 3+ bedroom dwelling ≥2.4 m (BADS)			✓					•						
40)	% of apartments that meet the minimum courtyard depth requirement based on the number of bedrooms and bathrooms: 1-bedroom ≥2 m; 2-bedroom ≥2 m; 3-bedroom ≥2.4 m (SEPP65/SPP7.3)	✓	✓						•						
41)	% of apartments that meet the minimum courtyard depth requirement based on the number of bedrooms and bathrooms: Studio or 1-bedroom ≥1.8 m; 2-bedroom dwelling ≥2 m; 3+ bedroom dwelling ≥2.4 m (BADS)			✓					•						
42)	Length (m) of the longest side of the communal outdoor space	✓	✓							•					
43)	Minimum width (m) of the communal outdoor space	✓	✓							•					
44)	% of the communal open space perimeter that is overlooked by apartment balconies	✓	✓	✓						•					
45)	% of the complex site area that is communal open space (standard ≥25%)	✓								•					
46)	Area of communal outdoor space provided is 6 m^2^ per apartment		✓							•					
47)	Area of communal outdoor space is 250 m^2^ or 2.5 m^2^ per apartment			✓						•					
48)	% of the communal open space that is grass		✓							•					
49)	Area (m^2^) of hardscaped (concrete, paving, decking) communal open space per apartment		✓							•					
50)	% of communal outdoor space area that is hardscaped (concrete, paving, decking)		✓							•					
51)	% of apartments located on a floor with ≤8 units	✓									•				
52)	% of apartments located on a floor with 9–12 units	✓									•				
53)	% of apartments located on a floor with ≤12 units		✓								•				
54)	% of apartments located on a floor with a window in the common corridor	✓	✓	✓							•				
55)	% of apartments located on a floor where the length of the longest straight run of the corridor (from the lift core) is ≤ 12 m	✓	✓								•				
56)	% of apartments located on a floor that meets the minimum corridor width (1.5 m) requirement		✓								•				
57)	% of apartments with balcony setbacks of ≥6 m from adjacent sites		✓									•	•		
58)	% of apartments with ≤50% of all bedrooms accessible directly off the living area	✓	✓					•				•			
59)	% of apartments where only one bathroom is directly accessible off the living area	✓	✓					•				•			
60)	% of apartments where the living room window does not open directly into external common circulation spaces	✓	✓									•	•		
61)	% of apartments where the main bedroom window does not open directly into external common circulation spaces	✓	✓									•	•		
62)	% of apartments where the living area is separated from external circulation spaces by service areas	✓	✓	✓								•			
63)	% of apartments where the main/1st bedroom is separated from external circulation spaces by service areas	✓	✓	✓								•			
64)	% of apartments where the number of party walls is limited to ≤2 side neighbours	✓		✓								•			
65)	% of apartments in the building that were assigned specific car parking bay/s	✓	✓											•	
66)	Number of visitor parking bays		✓											•	
67)	Number of motorbike/scooter parking bays	✓	✓											•	
68)	Area (m^2^) of bicycle parking	✓	✓											•	
69)	% of two-storey apartments	✓	✓												•
70)	% of mezzanine apartments	✓	✓	✓											•
71)	% of courtyard or terrace apartments	✓	✓	✓											•
72)	% of open plan (living/dining/kitchen area) apartments	✓	✓	✓											•
73)	% of studio apartments	✓	✓	✓											•
74)	% of 1-bedroom apartments	✓	✓	✓											•
75)	% of 2-bedroom apartments	✓	✓	✓											•
76)	% of 3-bedroom apartments	✓	✓	✓											•
77)	% of 4-bedroom apartments	✓	✓	✓											•
78)	% of apartment types (studio, 1-bed, 2-bed, 3-bed, 4-bed) in the building	✓	✓												•
79)	Entropy score for a mix of apartment types (higher levels of entropy = higher mix level)	✓	✓												•
80)	% of floors with ≥2 apartment types		✓												•

### Identifying cluster-derived building types and combinations of design features

Cluster analysis is an exploratory data analysis technique for organising observed data into meaningful groups based on combinations of independent variables, which maximises the similarity of cases within each group while maximising the dissimilarity between initially unknown groups.^22^ First, a Ward's hierarchical cluster analysis was employed to help determine the number of clusters. Hierarchical clustering typically starts with single objects which are combined to create clusters based on similarity. Clusters are then combined until one single cluster is achieved. The appropriate number of clusters is determined using the distance measure between cluster formations from the agglomeration schedule, where a large jump in the agglomeration schedule is indicative of the appropriate number of clusters. The K-means method is the most common of the 'partitioning' clustering analyses for splitting a dataset into a set of *K* groups.^22^ A series of *K*-means cluster analyses were run on all *n* = 80 measured design requirements for the *n* = 172 buildings to identify homogeneous clusters of buildings with differing combinations of design requirements. All variables were standardised (i.e., z-scores) so that each design requirement is reflected in the cluster analysis on the same scale. Different cluster solutions were obtained, and the within-cluster variance was used to decide on the optimal number of clusters (i.e., the number at which any further increase in clusters produced only a marginal reduction in the within-cluster variance).

Once the clusters were determined (*n* = 2), T-tests were run on the *n* = 80 design requirements (unstandardised variables) to determine statistically significant differences between apartment and building measures by cluster to assess how statistically distinct the clusters were from one another and identify which classifying variables were significantly different between the cluster groupings (i.e., which of the policy derived design features contributed most to the cluster solution).

### WEMWBS–positive mental health

Residents' positive mental health was measured using the Warwick–Edinburgh Mental Well-being Scale (WEMWBS).^23^ This scale was developed to measure mental wellbeing, investigate determinants of mental wellbeing in the general population, and evaluate projects, programmes and policies which aim to improve mental wellbeing.^23^ Participants responded to 14 items on a 1–5 Likert scale relating to their mental wellbeing (thoughts and feelings) in the previous two weeks. The items are worded positively to cover both feeling and functioning aspects of mental wellbeing including optimism, perception of usefulness, confidence, social interaction, energy and interest in new activities.^23^ The continuous scale was scored by summing the responses to each item answered (range: 14–70), with higher scores representing higher levels of positive mental well-being.

### Statistical analysis

All analyses were conducted in SPSS® Statistical package (version 27). All participants were assigned the cluster number of the apartment building in which they lived. Participants' sociodemographic characteristics, as well as the mean WEMWBS score of participants in the different clusters, were compared using Pearson's chi-squared tests (χ2) for categorical variables and independent samples t-tests for continuous variables. The differences in WEMWBS scores of participants in the two clusters was also estimated using linear mixed-effects models that controlled for sex, age, living with a partner, children living at home, employment status, household income, education level, self-rated general health status,^24^ length of residence, city (Perth, Melbourne, Sydney), area level disadvantage using the Australian Bureau of Statistics Socio-Economic Indexes for Areas (SEIFA) Index of Relative Socio-economic Disadvantage (IRSD) decile rankings (stratified into three groups: deciles 1–4 = high area disadvantage; deciles 5–7 = mid area disadvantage; and deciles 8–10 = low area disadvantage), self-selection factors and clustering of participants within buildings. Self-selection factors were derived from survey questions on the three factors influencing their choice of apartment: (1) apartment spaciousness, (2) natural light, and (3) natural ventilation to the apartment.^25^

### Role of the funding source

The project funders had no role in the study design, data collection, data analysis, interpretation or writing of the report. The Department of Planning Lands and Heritage (WA), Office of the Government Architect (WA), Government Architect NSW (GANSW), Planning Institute of Australia (PIA) and Development WA were study collaborators who provided in-kind support.

## Results

Table 2 presents the results of the cluster analysis and the mean values for each design requirement (n = 80) of the buildings in the two clusters. The distinguishing characteristics (i.e., significantly different design requirements) of the two identified building clusters are highlighted.

**Table 2:** Cluster-derived apartment development types.




Denotes the building cluster with significantly greater implementation of the design requirement.

Denotes the policy contains the significant design requirement after Bonferroni adjustment (p < 0.0006) from the “high policy performance buildings”.

Denotes the policy contains the significant design requirement (p < 0.05) from the “high policy performance buildings”**.**

Denotes the policy contains the design requirement.

^**†**^ Significant after adjusting for multiple tests (Bonferroni correction: p < 0.0006).

Cluster #1 buildings (*n =* 89) were characterised by having a statistically significantly greater implementation of *n* = 51 of the design requirements across nine design elements and were labelled the ***"high policy performance buildings".*** For a small number of these design factors, a lower value actually denoted more ‘positive’ design (e.g., a building having fewer single-aspect apartments; or building types that were smaller in scale, having fewer apartments, fewer buildings per complex, fewer floors, and smaller plot ratios). Apartments were characterised by higher levels of implementation of the solar and daylight access and ventilation design features (e.g., higher proportions of apartments with dual aspects, windows being present in all habitable rooms, and ratios of openable living room window areas to the open plan floor area being at least 5%). Additionally, apartments had a significantly higher implementation of design features for improving acoustic and visual privacy, including building separation standards and separation of living spaces and bedrooms from common and external circulation spaces. Cluster #1 buildings also had larger apartments, with a greater number meeting the minimum apartment, bedroom and private open space size and dimension standards and having more private storage areas external to the apartment. Further, these buildings provided larger communal outdoor spaces (with higher proportions of these spaces being grassed areas) and had more circulation spaces that met the corridor width, length, and the number of apartment requirements per floor. Finally, cluster #1 buildings provided higher levels of resident and visitor car parking and a greater mix of apartment types.

After a conservative multiplicity (Bonferroni) correction to account for the number of T-tests, *n* = 29 design requirements remained significantly different between the clusters at the p < 0.0006 alpha level (i.e., 0.05 alpha level ÷ number of variables/T-tests), indicating greater implementation of these design requirements compared with the second cluster group of buildings.

Cluster #2 buildings (*n* = 83) were named the ***"low policy performance buildings"***. They were characterised by significantly lower implementation of the apartment design policy requirements, performing worse than cluster #1 buildings across most design elements. These buildings also had more buildings within each development/complex and higher plot ratios (i.e., larger complexes) and had the highest proportions of single-aspect apartments.

Table 2 also indicates the source policies for each of the significantly different cluster-derived design requirements. Of the *n* = 51 significant design requirements (p < 0.05) from the *“high policy performance buildings”*, *n =* 38 were included in SEPP65 (NSW), *n =* 43 in SPP7.3 (WA) and *n =* 20 in BADS (VIC). Using the conservative Bonferroni adjusted alpha level (p < 0.006), of the *n* = 29 significant design requirements (p < 0.0006), *n* = 19 were included in SEPP65 (NSW), *n* = 25 in SPP7.3 (WA) and *n* = 11 in BADS (VIC).

Table 3 presents the characteristics of the High Life participant sample (*n* = 1135). The average age of participants was 42 years; the majority were female (61%), half (52%) lived with a partner and the majority (88%) had no children living at home, 50% were owner occupiers and 51% rented their apartment. Participants in the *"high policy performance buildings"* were significantly older (45 years of age) than those in the *"low policy performance buildings"* (40 years of age) and were more likely to be owner occupiers than renters. Further, Melbourne-based residents were much more likely to be in *“low policy performance buildings”* than Sydney or Perth-based residents, as were building residents located in neighbourhoods of mid-level area disadvantage (compared with those located in low disadvantage and, surprisingly, high disadvantage areas). There was also a significant difference in the WEMWBS scores of participants in cluster #1 buildings (51.8) versus those in cluster #2 buildings (49.8) (Table 3). Finally, Table 4 presents the results of the regression analysis. Residents in the *"high policy performance buildings"* had significantly higher (+1.96 points) WEMWBS (i.e., positive mental wellbeing) scores compared with residents in the *"low policy performance buildings".*

**Table 3:** Characteristics of the High Life participant sample.

	Overall % (n)	Cluster 1: High policy performance buildings % (n)	Cluster 2: Low policy performance buildings % (n)	P-value
**% (n)**	100 (1135)	37·8 (429)	62·2 (706)	
**Sex**				
Male	39·0 (443)	40·8 (175)	38·0 (268)	0·343
Female	61·0 (692)	59·2 (254)	62·0 (438)	
**Age**^a^	42·1 (15·7)	45·6 (17·0)	40·0 (14·4)	**<0**·**001**
**Living with partner**				
Partner	51·5 (584)	53·1 (228)	50·4 (356)	0·374
No partner	48·5 (551)	46·9 (201)	49·6 (350)	
**Children living at home**				
Yes	12·1 (137)	14·2 (61)	10·8 (76)	0·083
No	87·9 (998)	85·8 (368)	89·2 (630)	
**Housing tenure**				
Public housing	3·3 (37)	2·3 (10)	3·8 (27)	**<0·001**
Private rental	47·3 (536)	37·1 (159)	53·4 (377)	
Own outright or mortgage	49·5 (561)	60·5 (259)	42·8 (302)	
**Education**				
Secondary or less	13·8 (157)	15·2 (65)	13·0 (92)	0·153
Trade/certificate	19·3 (219)	21·4 (92)	18·0 (127)	
Bachelor or higher	66·9 (759)	63·4 (272)	69·0 (487)	
**Household income**				
$0–$60,000	23·7 (269)	23·8 (102)	23·7 (167)	0·466
$60,001–$100,000	24·4 (277)	21·9 (94)	25·9 (183)	
>$100,001	48·2 (547)	50·3 (216)	46·9 (331)	
Not reported	3·7 (42)	4·0 (17)	3·5 (25)	
**Employment status**				
Full-time employment	63·3 (719)	59·9 (257)	65·4 (462)	0·164
Part-time employment	16·4 (186)	16·6 (71)	16·3 (115)	
Not in paid employment	19·6 (222)	22·8 (98)	17·6 (124)	
Not reported	0·7 (8)	0·7 (3)	0·7 (5)	
**Self-rated general health**				
Poor	2·4 (27)	2·1 (9)	2·5 (18)	0·233
Fair	9·7 (110)	7·9 (34)	10·8 (76)	
Good	30·0 (341)	29·8 (128)	30·2 (213)	
Very good	43·9 (498)	47·6 (204)	41·6 (294)	
Excellent	14·0 (159)	12·6 (54)	14·9 (105)	
**Length of residence**^a^	2·2 (2·3)	2·2 (1·5)	2·3 (2·5)	0·558
**City**				
Perth	44·8 (508)	56·9 (244)	37·4 (264)	
Melbourne	33·4 (379)	2·6 (11)	52·1 (368)	**<0**·**001**
Sydney	21·9 (248)	40·6 (174)	10·5 (74)	
**Area disadvantage**				
IRSD decile 1–4 (High disadvantage)	24·5 (278)	31·2 (134)	20·4 (144)	
IRSD decile 5–7	35·6 (404)	25·9 (111)	41·5 (293)	**<0**·**001**
IRSD decile 8–10 (Low disadvantage)	39·9 (453)	42·9 (184)	38·1 (269)	
**Resident’ dwelling priorities/self-selection factors**^a^				
Apartment spaciousness	4·2 (0·7)	4·2 (0·7)	4·2 (0·7)	0·096
Natural light to the apartment	4·2 (0·8)	4·2 (0·8)	4·2 (0·8)	0·374
Natural ventilation to the apartment	4·1 (0·8)	4·2 (0·8)	4·0 (0·8)	**0·008**
**Mental wellbeing (WEMWBS)**	50·5 (8·7)	51·8 (8·9)	49·8 (8·5)	**<0**.**001**

P comparing differences from Pearson Chi-Square (categorical variables) and independent samples t-test (continuous variables). Bold denotes significant at p < 0.05.

a Mean and standard deviation (SD) for continuous variables.

**Table 4:** Positive mental wellbeing (WEMWBS) estimates by building cluster type.

	Estimate (SE)^a^	*p*
**Cluster 1:** High policy performance buildings	1·96 (0·59)	**0·001**
**Cluster 2:** Low policy performance buildings	0·00 (ref. group)	–
**Sex**		
Male	−0·82 (0·48)	0·088
Female	–	–
**Age**^b^	0·06 (0·02)	**0·001**
**Living with a partner**		
Partner	2·31 (0·49)	**<0·001**
No partner	–	–
**Children living at home**		
Yes	0·30 (0·72)	0·682
No	–	–
**Education**		
Secondary or less	−1·43 (0·72)	**0·047**
Trade/Certificate	−1·34 (0·62)	**0·030**
Bachelor or higher	–	–
**Household income**		
Not reported	1·67 (1·26)	0·185
$0–$60,000	−0·40 (0·71)	0·572
$60,000–$100,000	−0·22 (0·59)	0·712
>$100,000	–	–
**Employment status**		
Not reported	6·22 (2·81)	**0·027**
Full-time employment	0·37 (0·75)	0·623
Part-time employment	0·05 (0·82)	0·952
Not in paid employment	–	–
**Self-rated general health**		
Poor	−17·39 (1·64)	**<0·001**
Fair	−10·32 (0·98)	**<0·001**
Good	−6·96 (0·75)	**<0·001**
Very Good	−4·68 (0·71)	**<0·001**
Excellent	–	–
**Length of residence**^b^	0·00 (0·11)	0·973
**City**		
Perth	1·37 (0·66)	**0·042**
Melbourne	1·33 (0·79)	0·095
Sydney	–	–
**Area disadvantage**		
IRSD 1–4 (high disadvantage)	−0·46 (0·63)	0·473
IRSD 5–7	0·20 (0·57)	0·728
IRSD 8–10 (low disadvantage)	–	–
**Resident’s dwelling priorities/self-selection factors**^b^		
Apartment spaciousness	0·54 (0·33)	0·103
Natural light to the apartment	0·85 (0·41)	**0·039**
Natural ventilation to the apartment	0·04 (0·38)	0·911

Adjusted for sex, age, living with partner, children living at home, household income, education level, employment status, self-rated general health, length of residence, city, area disadvantage, residents' dwelling priorities/self-selection factors (apartment spaciousness; natural light; and natural ventilation to the apartment) and clustering within building. Bold denotes significant at p < 0.05.

a SE: standard error.

b Continuous variable.

## Discussion

Unprecedented global population growth has highlighted the need to consider how cities are built, and people are housed. As a result, Australian cities are experiencing a pronounced increase in apartment construction. The evidence to date suggests that housing design can affect the mental health of building occupants. Still, few studies have explored how to optimise the design of high-density housing to promote residents’ mental wellbeing.^26^ Consequently, there is a need for empirical evaluation of the design policies recently introduced in Australia to assess the performance of the delivered buildings. This can guide evidence-informed architectural and design policy and advance future practices for good apartment design and high-rise living.

This is the first study to examine: (1) the implementation and composition of design requirements derived from apartment design policies; and (2) their association with residents' positive mental health based on a substantial sample of residents and buildings across multiple cities. The results revealed a strong dose–response relationship between policy implementation and mental wellbeing. Residents living in ***‘high policy performance buildings’***, with greater implementation of a combination of design requirements (*n* = 29) for solar and daylight access, acoustic and visual privacy, private open space, storage, communal circulation spaces, car parking and greater mixes of apartments, had significantly higher positive mental wellbeing.

The key design requirements identified in this study are supported by previous studies on housing design and health. The benefits of natural light are well documented, from vitamin D production (a lack of which has been associated with depression and obesity) to enhancing sleep patterns, mood, focus and productivity.^6^ Inadequate daylighting or poor window views have been found to increase the probability of depression by 60% and 40%, respectively.^3^^,^^6^ Our findings also align with studies on indoor environments and the importance of natural ventilation and thermal comfort on occupants' physical health and cognitive function.^3^^,^^6^ While noise is pervasive in urban environments,^27^ noise from neighbours is perceived as more annoying by apartment residents and has been linked to a range of non-auditory health effects and adverse health outcomes,^27^ including sleep disturbance, cardiovascular disease and impairment of cognitive performance in children.^28^ Perceptions of apartment space and layout, and communal area quality have also been independently associated with mental wellbeing.^3^^,^^8^ Internal private space typically impacts wellbeing via crowding, but quality communal areas may help minimise crowding by providing exposure to green space and the opportunity to interact with neighbours.^8^ An analysis of the communal outdoor space in the High Life buildings found the use of outdoor areas was positively associated with neighbouring, which protects against loneliness,^29^ and in turn, poor mental health.^30^ Importantly, our study approached apartment design holistically, testing a wide range of requirements across multiple design elements to identify the combination of requirements that are associated with mental health, rather than a siloed focus on specific design elements.

Our study derived measures from three operational apartment design policies, but notably these policies provided different levels of design guidance, as evidenced by the different number of design requirements represented from each state policy (Table 2). For example, of the *n* = 29 design requirements in Cluster #1 that were positively associated with increased positive mental health scores using the more stringent Bonferroni significance level, *n* = 11 (37.93%) are currently included in the Victorian government design policy (BADS). This suggests that in its current form, the policy may be unable to bring about positive mental health benefits. Conversely, the NSW (SEPP65) and WA (SPP7.3) policies contained *n* = 19 (65.52%) and *n* = 25 (86.21%) of the identified optimal mix of design requirements, respectively. These results provide clear evidence to help policymakers advocate for the adoption or inclusion of specific requirements (where currently missing) or the retention of design requirements (where presently included) during future policy reviews and iterations. Indeed, several design requirements that were included in the original draft of the Victorian standards were relaxed or removed after public comment,^16^ highlighting the importance of empirical evidence to support or defend key standards and policy decisions.

This study has several strengths that uniquely contribute to the evidence base. First, interdisciplinary collaborations that integrate social scientists, health researchers, urban designers and architects are scarce. However, they are vital to facilitate meaningful research exploring the relationship between apartment design and mental health and isolate design factors' effect on mental health.^6^ This study directly addresses this gap through a unique multidisciplinary and rigorous approach that provides policymakers, architects, and urban designers with empirical evidence on apartment design policy implementation and its association with mental health in three Australian capital cities. Second, a novel aspect of the High Life project was measuring the on-ground delivery of the apartment design policies in the buildings (i.e., the 'dose' of the policy intervention that had been delivered). The development and use of policy-specific and architecturally grounded measures of design in apartment buildings is unique. Third, using cluster analysis to characterise the buildings based on the combination of the design features implemented is a novel approach to quantify the design performance of the apartment buildings.

Lastly, research (in adults) suggests that the WEMWBS could detect a clinically meaningful change.^31^^,^^32^ A review of 12 prior studies tested whether WEMWBS was able to detect a change on mental wellbeing at a group level.^31^ Across all studies the standardised response mean (calculated by dividing the mean change in score by the standard deviation of the change score) was greater than 0.5 (ranging from −0.10, 95% CI: −0.35, 0.15 to 1.35, 95% CI: 1.06, 1.64) which compares favourably to other mental illness and life satisfaction scales, generic health-related quality of life scales, and disease specific scales and indicates that WEMWBS is responsive to changes in mental health interventions in different populations^31^ Our mean difference in cluster groups of 2 points (51.8 *"high policy performance buildings"*, 49.8 *"low policy performance buildings"*) and a regression estimate of a 1.96 higher WEMWBS score in *"high policy performance buildings"* versus *"low policy performance buildings"* indicates a sufficiently large difference between the clusters to be of real and meaningful difference in mental wellbeing of the two clustered groups.

The three design policies evaluated are performance-based, thus developers are not required to meet all standards if they apply innovative solutions that satisfy the intent of the objectives.^17^^,^^18^ Indeed, our previous analysis found that buildings in Sydney (NSW) had on average implemented just 57% of the measured NSW policy design requirements.^3^ The results of this study identified the combinations of policy design requirements that architects were adopting. Notably, the findings indicate that when this combination of selected design requirements is implemented to a sufficient level, the policy can be a viable intervention to promote the positive mental wellbeing of occupants.

While this study focused on Australian apartment design policies, the results are directly applicable to other international policies that were similarly introduced to regulate the standard of high-rise buildings and apartment design in their respective jurisdictions.^33^, ^34^, ^35^ These findings provide empirical evidence for the inclusion or retention of specific design features and architectural solutions in future iterations of the policies for promoting mental wellbeing.

This study makes a unique contribution to the evidence base. However, it has some limitations: (1) the cross-sectional design means causality cannot be inferred; (2) The design requirement standards are based mainly on industry 'best practice' and intuition rather than empirical evidence, and there has been little evaluation of whether these thresholds are appropriate. Testing the appropriateness of the (minimum) policy standards was outside the scope of this study but is an important area for future research; (3) Building measures were extracted from the architectural plans and elevations submitted in the development application process. Whilst it is possible that the final constructed buildings deviated from the approved development application materials, our measurement process screened the building plans against other data sources (e.g., Strata title information, real estate listings, marketing materials) and buildings that were noticeably different from the approved plans were excluded from the study^3^; (4) Our analysis controlled for numerous socio-demographic variables associated with mental health, however, we accept that mental health may be impacted by other, unmeasured confounders, including the location of the apartment building, levels of crime and violence in the neighbourhood and perceived neighbourhood disorder (e.g., vandalism, lack of facilities, vacant housing and litter) and air pollution.^11^ (5) Human ethics approval for the study required the focus of the survey to be stated on all recruitment materials. As such participants may have been able to surmise the study hypothesis which may have subconsciously biased them to respond in a way they think is expected.^36^

### Conclusion

This study addresses a significant gap in the literature, providing empirical evidence that apartment buildings developed in accordance with Australian apartment design policy requirements have the potential to promote the positive mental wellbeing of the inhabitants. Our results have practical implications as they identify the combination of design requirements that should be prioritised in building approval processes to promote optimal resident mental health. The findings reiterate the importance of architecture and design instruments that facilitate the implementation of minimum policy standards to guide architectural and urban design thinking, policy, and practice and, ultimately, the health of future high-rise housing stock.

## Contributors

SF conceived and designed the study. PH developed the measures with input from SF, NE and JB. AK conducted the analyses, and PH drafted the manuscript. All authors contributed to the manuscript drafts and read and approved the final manuscript.

## Data sharing statement

Data collected for the study can be made available on request from the authors.

## Declaration of interests

All authors have completed the ICMJE uniform disclosure form at www.icmje.org/coi_disclosure.pdf and declare: no disclosures, except for SF who reports payment or honoraria by: Environment & Behavior for services as Assistant Senior Editor; Department of Environment, Land, Water and Planning (DELWP) for participation in a panel; Government of Western Australia for participation as a member of State Design Review Panel; and the Conference of the International Association of People- Environment Studies for a conference keynote presentation.
